# Editorial: Functional Genomics in Plant Breeding 2.0

**DOI:** 10.3390/ijms23136959

**Published:** 2022-06-23

**Authors:** Fatemeh Maghuly, Eva M. Molin, Rachit Saxena, David J. Konkin

**Affiliations:** 1 Plant Functional Genomics, Institute of Molecular Biotechnology, Department of Biotechnology, BOKU-VIBT, University of Natural Resources and Life Sciences, 1190 Vienna, Austria; 2 Center for Health & Bioresources, AIT Austrian Institute of Technology (AIT), 3430 Tulln, Austria; eva-maria.molin@ait.ac.at; 3 The International Crops Research Institute for the Semi-Arid Tropics (ICRISAT), Patancheru 502324, India; rachitsaxena@gbu.edu.in; 4 Aquatic and Crop Resource Development (ACRD), National Research Council Canada (NRC), Saskatoon, SK S7N 0W9, Canada; david.konkin@nrc-cnrc.gc.ca

Scientists agree that the increased human impact on the environment since the 19th century has positioned our planet in a period of rapid and intense change, particularly to our natural ecosystems [1]. Most plants suffer from physiological and biochemical damages upon exposure to increased biotic and abiotic stresses. Regarding crops, stress-induced injuries, which are reflected in most metabolic processes, can cause substantial losses in yield [2]. Crop species are equipped with vast diversity in stress adaptation mechanisms. Although many of these mechanisms are universal, their relative importance may vary from genotype to genotype. Consequently, some genotypes can cope with stress better than others [3,4]. Genotypes that differ in stress adaptation/acclimation mechanisms serve as an essential resource for studying these mechanisms. Based on available genotypic variability and given the considerable impact of genomic structural variation on gene function, we need to increase our understanding of the genome and its relationship with the plant phenotype related to the trait of interest.

Thus, one of our goals is to understand the molecular mechanisms underlying how plants respond to various stresses to regulate their development and immunity. Proper responses to environmental signals are essential for optimal growth, reproduction, and fitness. Understanding their molecular basis is fundamental to the central biological question of signaling and adaptation, and better prepares us for global climate change. The characterization of gene function and the key regulatory genes’ response to external and internal stresses is the next focus to identify endogenous genes whose expression provides interesting phenotypic signatures.

Furthermore, identifying the distinct expression levels of related genes will open up significant opportunities for correlating these expression patterns related to the desired trait of interest using integrative analyses. Moreover, to successfully benefit from the obtained data, it is necessary to address further aspects of the interaction between genetics, the environment and its changing climate, and the phenotypes. Therefore, this Special Issue “Functional Genomics in plant Breeding 2.0”, with seven original articles and three reviews, contributes to a further understanding of the available information to identify, characterize and deliver novel candidate genes related to biotic and abiotic stresses, as well as important economic pathways. Furthermore, pan genomic approaches and the importance of a suitable reference genome adding functional priors to build genomic predictions for multi-trait genomic selection systems are discussed.

In this context, the construction of reference genome assemblies for crop species significantly improved the ability to associate traits with genomic regions, increasing the success of the selection of agronomically beneficial phenotypes [5].

As foundational resources to support functional, comparative and molecular research into crop plants for sustainable agriculture, particularly legume community research, Kaur et al. [6] reported significantly improved draft genomes of *Medicago truncatula*, a model legume for genetic study. They used a Hi-C contact map to anchor, order, orient scaffolds, and correct contigs of the previously published genome assembly R108 v1.0 [7]. Their improved genome assembly contains eight chromosome-length scaffolds that span 97.62% of the sequenced-based assembly.

However, using a single reference genome is not sufficient to represent the entire genetic diversity due to the significant structural variation (SV) observed within a species. On the other hand, pangenomes are references that capture the genetic diversity of a species, allowing for a more accurate prediction of traits [8,9]. Unfortunately, pangenome data are not available for many less prominent crop species, being essential food sources in regional populations. Here, Tay Fernandez et al. [5] reviewed the pangenome approaches of several under-utilized crops cultivated in arid or semi-arid environments. Their newly characterized genes related to drought tolerance could build resources for improvement and introgression into major crop species.

Given the significant decreases in yield quantity and quality of crops worldwide due to fungal diseases and the economically unjustified and potentially harmful application of fungicides in the environment, Toporowska et al. [10] investigated the identification, localization, and validation of a new source of monogenic crown rust resistance in oats. Although the effectiveness of monogenic crown rust resistance genes can be overcome in a short time by the pathogen, it is a much easier and faster strategy than the introduction of permanent resistance genes. They also developed a linked PCR marker for the identified resistance gene (Pc50-5), useful for marker-assisted selection.

In addition, comparative transcriptome analysis of late leaf spot (LLS) caused by the fungus *Nothopassalora personata* in groundnut was studied by Gangurde et al. [11]. They presented new information on the candidate resistance genes with differential gene expressions at various disease and symptom development stages, including defoliation in groundnut. The available data in this study can be deployed to develop functional markers and genome editing to grow resistance cultivars.

Focusing on biotic resistance to the two most economically and agronomically harmful insect pests (wheat stem sawfly and orange wheat blossom midge), Muslu et al. [12] described a comparative analysis of coding and non-coding features of loci associated with resistance across important cereal crops, barley, rye, oat, and rice. They suggested that the identified microRNA (miRNA) may lead and contribute to plant resistance response, which can be used in insect resistance breeding.

To gain new insight into the molecular mechanisms underlying saccharification of sweet potato storage roots to understand how plants respond to high temperatures and to provide theoretical bases for the breeding of high-quality sweet potatoes, Li et al. [13] analyzed three high and low saccharified sweet potato cultivars to determine differentially expressed genes and metabolites under extreme conditions. The study provided novel insight into the important pathway for saccharification of sweet potato and delivered new candidate genes that can be used to improve and breed new table cultivars.

To fill the gap in our understanding of the genetic mechanisms underlying starch metabolism and its relationship with its physicochemical and functional properties, Yu et al. [14] summarized the starch biosynthetic pathway in peas and starch granule structure, and its connection with functional properties and industrial applications. They also described that the identification and characterization of novel genes and novel allelic variations in known genes might provide further gene targets for increasing our understanding of starch metabolism to design multifunctional native pea starch for custom modulating starch composition and structure and, thus, functionality.

It is generally acknowledged that the incorporation of genomic variants causing variation in the phenotypic trait can help to improve the performance of crop selection and yield improvement in cereal crops such as wheat ideotype. Thus, Jablonski et al. [15] reported the phenotypic effect of TaCKX1 and TaCKX2 silenced genes genotyping dependent in awned-spike cultivar, which differ in content and metabolism. They showed that those differentiations might influence the gene expression of silent genes, phytohormone regulation and yield-related traits. They also documented that some newly detected phytohormones such as phenylacetic acid (PAA) and toponis are specific to the awned-spike cultivar. The obtained data are important since the future wheat ideotype breeding program requires a more holistic approach than gene functional analysis.

In seed-bearing plants, the number of ovules determines the potential of seed number per fruit, significantly affecting the final seed yield; therefore, it is a significant research aspect of plant science. Hence, Qadir et al. [16] summarized the ovule number’s regulators, regulatory agencies, pathways, and molecular networks. This review also gives suggestions for the molecular improvement of ovule numbers, which will be very useful for plant breeders.

It is well known that one of the significant challenges in breeding hybrid wheat is the regulation of male sterility; furthermore, one of the most valuable strategies is the combination of the cytoplasmic male sterility (CMS) of the female parent with the fertility-restoring genes (dominant Rf alleles) of the male parent. In this regard, Tyrka et al. [17] provided integrative analyses of RNA-seq, PAG, and qRT-PCR to identify new Rf candidate genes and evaluate known Rf genes, which can be relevant for hybrid wheat breeding in restoring and non-restoring cultivars. They also suggested that the identified genes can serve as a source for developing molecular markers to select hybrids.

Here, we wish to thank all contributors to this Special Issue and hope that it will raise interest in and enhance understanding of functional genomics in plant breeding. Nonetheless, the current and previous [18] Special Issue can only cover a small part of our scope; therefore, we are pleased to represent the opening of the third part of this Special Issue, “Functional Genomics for Plant Breeding 3.0”, for which we invite our readers to follow and contribute. The new issue will continue to cover various research topics and review articles regarding recent development in omics platforms that can enhance breeding strategies to speed up the time and efficiency of the breeding process.
